# Deputy-Inspector-General John Jack

**Published:** 1910-09-17

**Authors:** 


Deputy-Inspector-General John Jack, R.N., the oldest surgeon on the retired list of the medical staff of the Service, has died at the age of ninety-two. He qualified as a licentiate of the Royal College of Surgeons of Edinburgh in 1839 and entered the Navy in 1842, becoming a staff surgeon in 1854 and a fleet surgeon in 1867. He retired in 1875. He gained a Sir Gilbert Blane Gold Medal, awarded to medical officers for the most approved journals of their practice while in medical charge of a man-of-war.

